# Supplementary material for: Application of Laser-Induced Breakdown Spectroscopy in the Quantitative Analysis of Elements—K, Na, Ca, and Mg in Liquid Solutions
Source: Materials (Basel). 2022 May 23;15(10):3736. doi: 10.3390/ma15103736 (PMC9146648; doi:10.3390/ma15103736)
Supplement: Supplementary file 1 [file materials-15-03736-s001.zip › materials-1663231-supplementary.pdf]

materials

Supplementary Mateials

Application of Laser-Induced Breakdown Spectroscopy in the Quantitative Analysis of Elements – K, Na, Ca and Mg in Liquid Solutions

Wojciech Skrzeczanowski * and Maria Długaszek

Military University of Technology, Institute of Optoelectronics, ul. gen. Sylvester Kaliski 2, 00-908 Warsaw 46, Poland; maria.dlugaszek@wat.edu.pl
* Correspondence: wojciech.skrzeczanowski@wat.edu.pl

Citation: Skrzeczanowski, W.; Długaszek, M. Application of Laser-Induced Breakdown Spectroscopy in the Quantitative Analysis of Elements – K, Na, Ca and Mg in Liquid Solutions. *Materials* **2022**, *15*, 3736. https://doi.org/10.3390/ma15103736

Academic Editor(s): Valery V. Tuchin

Received: 23 March 2022
Accepted: 18 May 2022
Published: 23 May 2022

Publisher's Note: MDPI stays neutral with regard to jurisdictional claims in published maps and institutional affiliations.

Copyright: © 2022 by the authors. Licensee MDPI, Basel, Switzerland. This article is an open access article distributed under the terms and conditions of the Creative Commons Attribution (CC BY) license (https://creativecommons.org/licenses/by/4.0/).

The results of the calibration for K, Na, Ca, and Mg are presented in Figures S1–S8. They are presented in 0–1000 µg/mL and 0–100 µg/mL ranges (0–250 µg/mL for K). As it is best seen for K calibration (Figure S1), the curve profiles in the 0–1000 µg/mL are not perfectly linear—they can be approximated by two straight lines—one for lower contents and the other one—for bigger concentrations. The low range curves are much steeper and their slopes are higher. That is why they were used for the determination of LODs (LOD = 3 σ/s, where σ is the background noise and s—is the curve slope). In all 8 cases coefficients of determination R^2 are between 0.944 for Na and 0.999 for Ca.

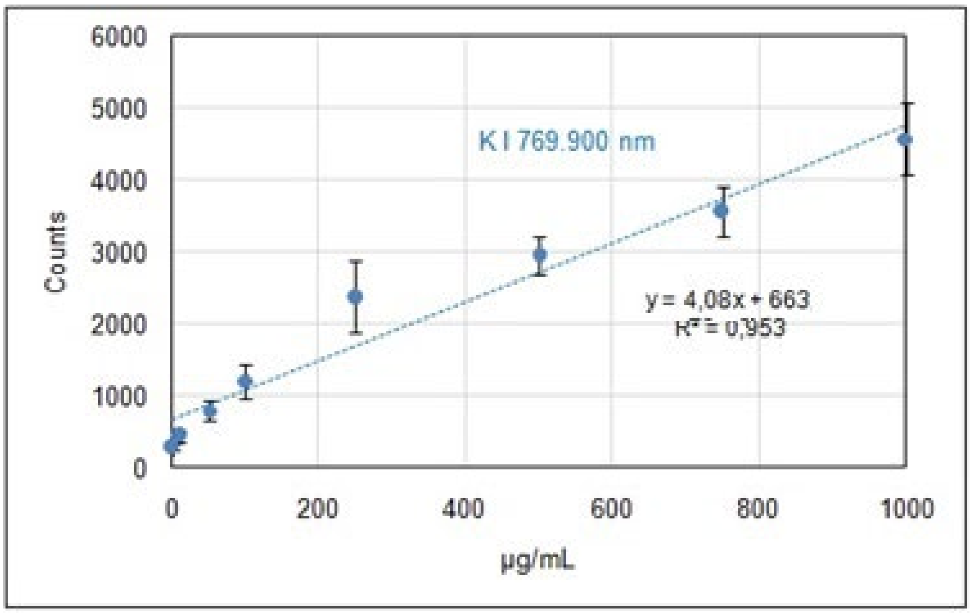

Figure S1. Calibration curve for potassium in the range 0–1000 µg/mL.

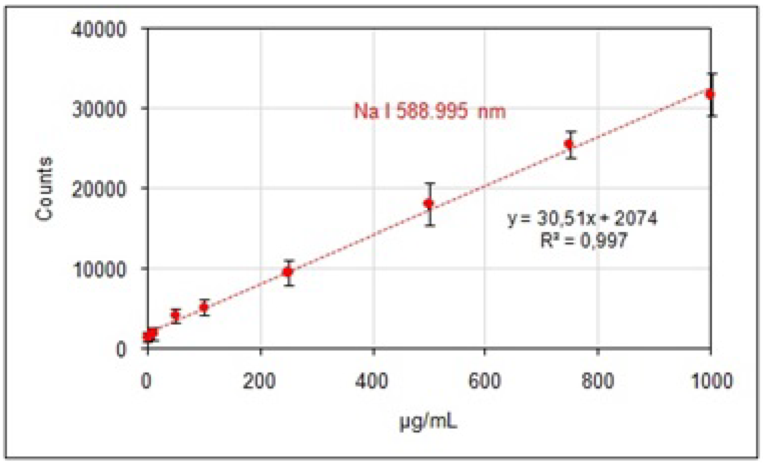

Figure S2. Calibration curve for sodium in the range 0–1000 µg/mL.

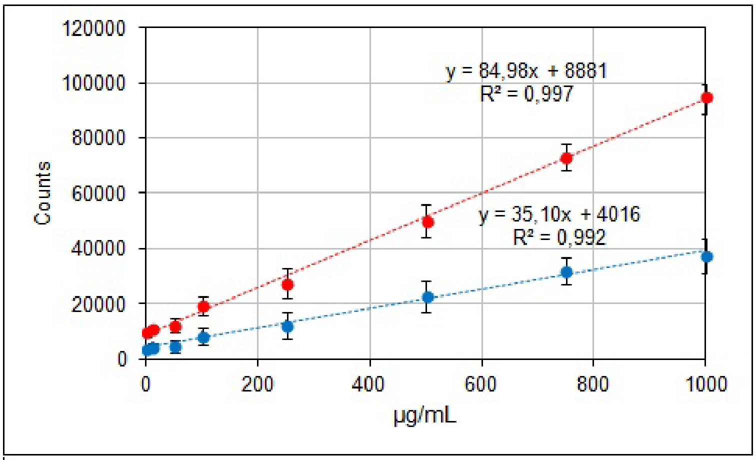

Figure S3. Calibration curve for calcium in the range 0–1000 µg/mL.

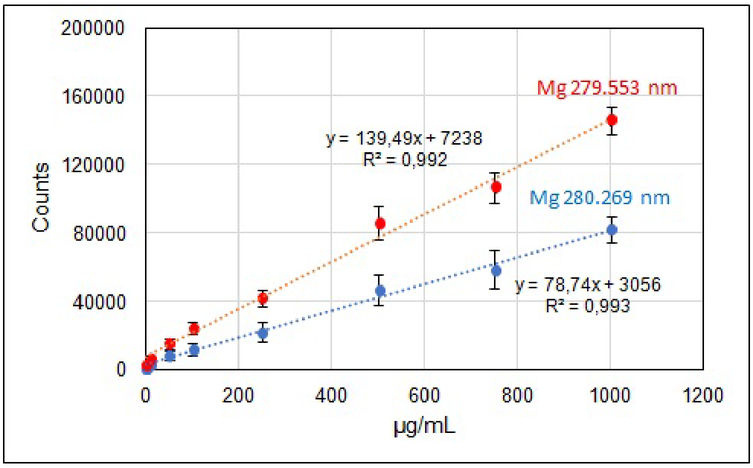

Figure S4. Calibration curve for magnesium in the range 0–1000 µg/mL.

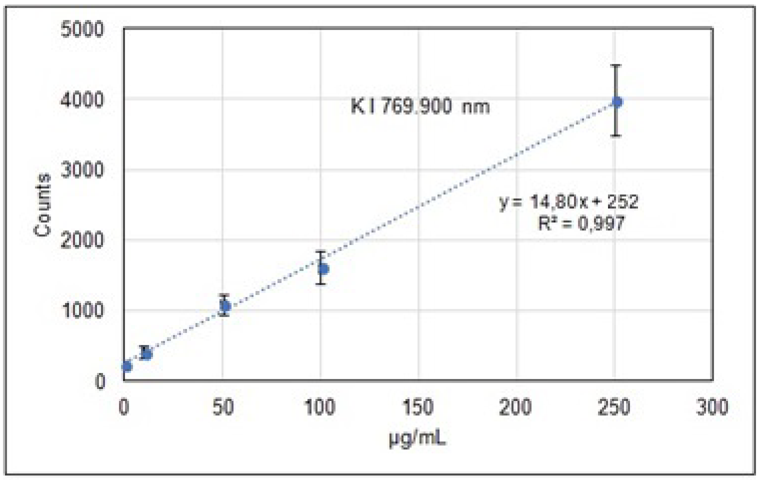

Figure S5. Calibration curve for potassium in the range 0–250 µg/mL.

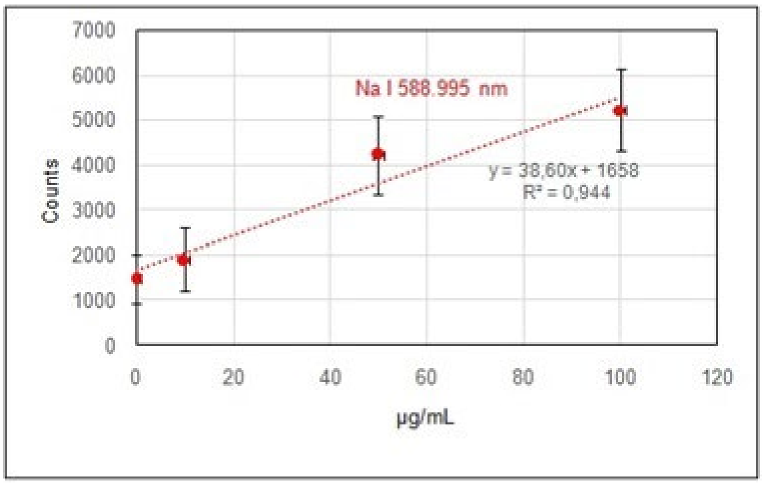

Figure S6. Calibration curve for sodium in the range 0–100 µg/mL.

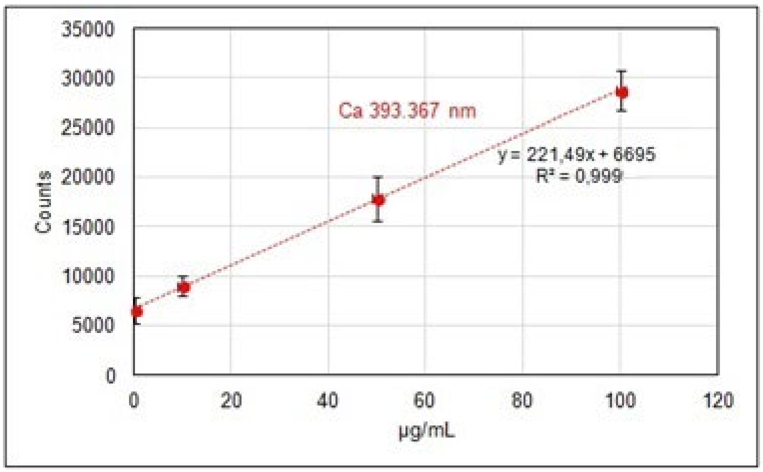

Figure S7. Calibration curve for calcium in the range 0–100 µg/mL.

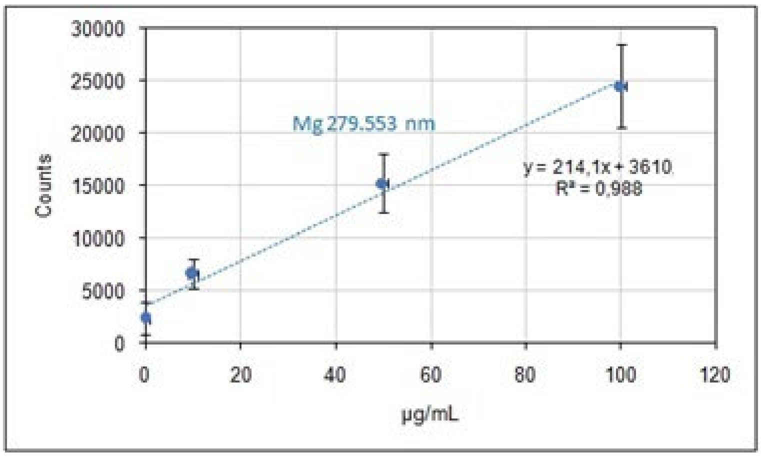

Figure S8. C alibration curve for magnesium in the range 0–100 μg/mL.

In Figure S9 an explanation of the selection of K I 769 nm line (instead of the stronger K I 766 nm line) is presented. Because of specific optical arrangement applied in the ESA 4000 spectrometer, for longer wavelengths "holes" in the spectra are observed. This deficiency is compensated by a very high spectral resolution of the instrument ($\lambda/\Delta\lambda$ ~ 20,000).

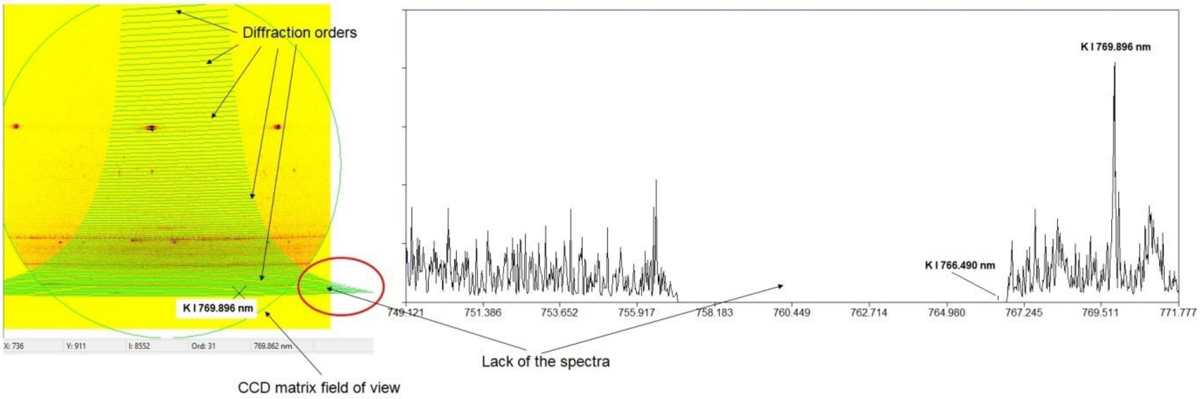

Figure S9. Illustration of the influence of the optical arrangement of the ESA 4000 spectrometer on spectra registration: left - small spectra gaps in the long-wavelength part of the spectral range (from about 550 nm up to 800 nm) resolved on the CCD matrix, right—a fragment of the registered spectrum in the vicinity of the two relatively strong atomic potassium K I 766.49 nm and K I 769.90 nm lines.

Exemplary spectra for 500 μg/mL of K, Na, Ca, and Mg samples, respectively, are shown in Figure S10.

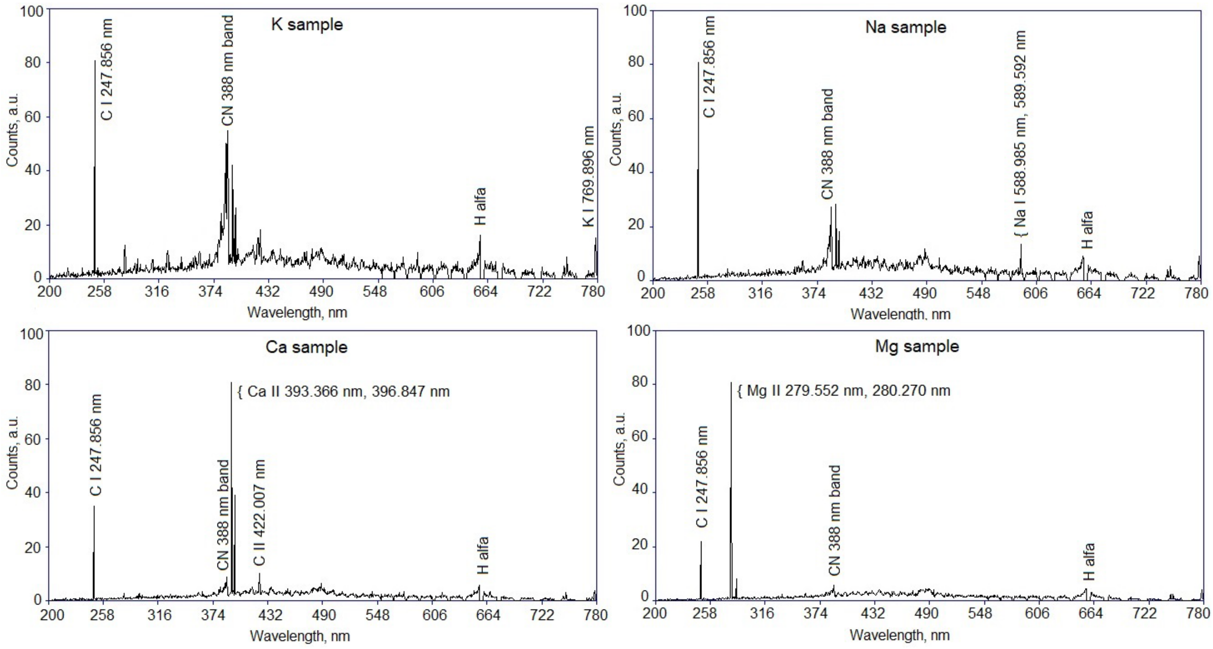

Figure S10. LIBS spectra for K, Na, Ca, and Mg samples with 500 µg/mL contents.
